# The safety and effectiveness of high-calorie therapy for treating amyotrophic lateral sclerosis: a systematic review and meta-analysis

**DOI:** 10.1007/s00415-023-11838-4

**Published:** 2023-06-28

**Authors:** Qiaochu Zhu, Dandan Xu, Hai Huang, Dong Li, Dan Yang, Jing Zhou, Yan Zhao

**Affiliations:** 1 College of Acupuncture and Orthopedics, Hubei University of Chinese Medicine, Wuhan, 430065 China; 2 First Clinical Medical College, Hubei University of Chinese Medicine, Wuhan, 430065 China; 3 Department of Tuina and Rehabilitation Medicine, Hubei Provincial Hospital of Traditional Chinese Medicine, Wuhan, 430061 China; 4 Department of Tuina and Rehabilitation Medicine, Affiliated Hospital of Hubei University of Chinese Medicine, Wuhan, 430061 China; 5 Department of Tuina and Rehabilitation Medicine, Hubei Institute of Traditional Chinese Medicine, Wuhan, 430061 China; 6 Department of International Culture Education, Chodang University, Muan, Republic of Korea

**Keywords:** Amyotrophic lateral sclerosis, High-calorie therapy, Nutrition, Systematic review, Meta-analysis

## Abstract

**Background:**

Amyotrophic lateral sclerosis (ALS) is a neurodegenerative disorder affecting the upper and lower motor neurons, which can lead to death from respiratory failure within 3–5 years after the onset of this disease. Nowadays, no drug can effectively slow down the progression of this disease. High-calorie therapy, an emerging complementary alternative treatment, has been reported in studies to prolong the survival time of patients, prevent muscle atrophy and provide a better prognosis. However, no systematic review and meta-analysis were performed to summarize the evidence of this therapy. This meta-analysis comprehensively evaluates the effectiveness and safety of high-calorie therapy for treating ALS.

**Methods:**

We searched the electronic databases from inception to 1 April 2023: PubMed, Embase, Web of Science, Cochrane Library, Scopus, Ovid/Medline, and ProQuest. Randomized controlled trials (RCTs) that met the inclusion criteria were performed by meta-analysis. All statistical analyses were performed in STATA software.

**Results:**

A total of six eligible RCTs were included in this meta-analysis, involving 370 ALS patients. The meta-analyses showed that high-calorie therapy had superiority in improving body weight (SMD = 1, 95% CI 0.36, 1.65) and BMI (SMD = 0.83, 95% CI 0.02, 1.63). With respect to safety, there was no difference between the high-calorie therapy and the control group regarding the number of adverse events (RR = 3.61, 95% CI 0.08, 162.49). However, ALSFRS-R scores (SMD = 0.34, 95% CI − 0.4, 1.08), survival rate (RR = 1.23, 95% CI 0.98, 1.55), and lipid profile (LDL: SMD = 0.21, 95% CI − 0.33, 0.75; HDL: SMD = 0.17, 95% CI − 0.37, 0.71; TC: SMD = 0.21, 95% CI − 0.33, 0.75), CRP (SMD = 0.85, 95% CI − 1.37, 3.06) showed no significant difference compared to the control groups.

**Conclusions:**

High-calorie therapy is effective in gaining weight and BMI with few side effects. However, no significant superiority was detected in ALSFRS-R scores, survival time, lipid profile, and CRP indicator. The overall quality of the included studies is high, and the results have some credibility, but future corroboration by high-quality RCTs is also expected.

## Introduction

Amyotrophic lateral sclerosis (ALS) is an incurable neurodegenerative disease affecting the upper and lower motor neurons in the spinal bulb, cerebral cortex, and spinal cord [1]. The clinical features of ALS are limb paralysis, muscle atrophy, dysphagia, dysarthria, and shortness of breath, which can lead to death due to respiratory failure [2]. According to the epidemiology of ALS, the incidence rate is estimated to be approximately 2.6 cases per 100,000 individuals annually [3], and the median survival period is only 30 months [4]. Nowadays, the etiology and pathogenesis of ALS remain unclear, and no effective cure has yet been found for this condition. Riluzole, a glutamatergic neurotransmission inhibitor, can slightly benefit the survival period and has been approved by the USA Food and Drug Administration (FDA). It is most commonly used in clinical practice [5]. However, an original cost-effective analysis found that the price range for riluzole fluctuated widely. ALS patients spend approximately $1000 per year on riluzole in the USA [6]. Nevertheless, in the UK, annual spending on the drug ranges from £834 to £3263 among ALS patients [7]. Overall, riluzole has been considered an expensive drug that imposes a significant financial burden on patients and families and can only prolong the life span for 3–5 months. In recent years, some emerging clinical investigational drugs, which the FDA also proved for the treatment of ALS, including relyvrio and in familial forms linked to SOD1 mutation, tofersen, have been able to alleviate disease progression to some extent. However, their effectiveness still needs to be corroborated by long-term follow-up and rigorously designed RCTs [8]. Considering the high expenses, a growing number of patients prefer palliative care and less-cost treatment.

Recently, weight loss has been considered as an independent prognostic factor for survival in ALS9. An observational study on malnutrition status during the disease found negative correlations between fluctuation in body mass index (BMI) and mortality [10]. The result showed a 30% increase in mortality for every one-point loss in BMI. The multicausal explanation of weight loss is that ALS patients with increasing resting energy expenditure exhibit a status of intrinsic hypermetabolism [11]. It has been proven that high-calorie nutrition can stabilize body weight. Targeting weight loss and supportive therapy for ALS patients may be effective, since increasing the calories in the diet of the ALS mice model showed positively prolonged survival [12] and a homogeneous trend in ALS patients with gastrostomy [13].

High-calorie diet therapy is an easy-to-implement, low-cost life intervention therapy. Compared to receiving treatment in professional medical institutions, lifestyle intervention has gained popularity among ALS patients worldwide for its advantages of being less painful and easier to accept. Therefore, researchers have shifted their focus to nutritional therapy to extend the survival period of ALS patients. High calorie includes a variety of nutritional therapies such as high-fat, high-carbohydrate, and high-protein nutrition [14]. Although this intervention can effectively stabilize body weight, there is no direct evidence showing that high-calorie therapies are safe and effective for ALS patients' survival period. Meanwhile, several studies have pointed out that this therapeutic intervention frequently accompanies a range of side effects [13, 14], such as gastrointestinal reactions and dysphagia. Evidence regarding high-calorie therapy's comparative safety and effectiveness remains uncertain. Given this scenario, evidence-based strategies are required to support judicious high-calorie therapy while ensuring the potential effects in ALS patients' management. Hence, we did a systematic review and meta-analysis to assess the safety and effectiveness of high-calorie therapy for ALS.

## Methods

This meta-analysis adhered to the Preferred Reporting Items for Systematic Reviews and Meta-Analyses (PRISMA) guidelines [15] and has been registered in PROSPERO (CRD42023414623). There is no need for ethical approval because this is a systematic review.

### Search strategy

We systematically searched the following electronic databases from inception to 1 April 2023: PubMed, EMBASE, Web of Science, Cochrane Library, Scopus, Ovid/Medline, and ProQuest. The search strategy used MeSH terms and keywords: ‘Amyotrophic Lateral Sclerosis,’ ‘energy intake,’ ‘Nutrition therapy,’ ‘randomized controlled trial,’ and ‘placebo.’ To ensure literature saturation, we comprehensively searched clinical trials, which are ongoing via the WHO International Clinical Trials Registry Platform (WHO ICTRP) and ClinicalTrials.gov. Preprint servers (such as medRxiv and Research Square) were searched for unpublished data. The search strategy of PubMed is shown in Table 1. The rest of the databases comply in the same way.

**Table 1:** Search strategy of PubMed

Search number	Query	Results
#1	"Amyotrophic Lateral Sclerosis"[Mesh]	22,994
#2	((((((((Sclerosis, Amyotrophic Lateral[Title/Abstract]) OR (Gehrig's Disease[Title/Abstract])) OR (Gehrig Disease[Title/Abstract])) OR (Charcot Disease[Title/Abstract])) OR (Motor Neuron Disease[Title/Abstract])) OR (Lou Gehrig's Disease[Title/Abstract])) OR (ALS—Amyotrophic Lateral Sclerosis[Title/Abstract])) OR (ALS Amyotrophic Lateral Sclerosis[Title/Abstract])) OR (Lou Gehrig Disease[Title/Abstract])	6322
#3	#1 OR #2	27,046
#4	"Nutrition Therapy"[Mesh]	112,956
#5	(((Diet, High-Fat[MeSH Terms]) OR (Diet, High-Protein[MeSH Terms])) OR (Energy intake[MeSH Terms])) OR (nutritional support[MeSH Terms])	118,934
#6	(((((((((Therapy, Nutrition[Title/Abstract]) OR (Medical Nutrition Therapy[Title/Abstract])) OR (Nutrition Therapy, Medical[Title/Abstract])) OR (Therapy, Medical Nutrition[Title/Abstract])) OR (Nutrition therapy[Title/Abstract])) OR (High-Fat Diet[Title/Abstract])) OR (Diet, High Fat[Title/Abstract])) OR (Diet, High Protein[Title/Abstract])) OR (Diets, High-Protein[Title/Abstract])) OR (High-Protein Diet[Title/Abstract])	41,825
#7	#4 OR #5 OR #6	193,484
#8	randomized controlled trial[Publication Type] OR randomized[Title/Abstract] OR placebo[Title/Abstract]	999,446
#9	#3 AND #7 AND #8	15

### Inclusion and exclusion criteria

The following studies were included if they met the inclusion criteria: (1) RCTs of high-calorie therapy for ALS; (2) RCTs with high fat, high protein, and high carbohydrate as components of high calorie were included; (3) patients diagnosed with ALS by a professional medical institution, regardless of gender, age, nationality, or race; (4) the experimental groups on either high-calorie therapy only or combined high-calorie therapy with other common treatment; (5) the control group had conventional therapy.

The exclusion criteria of the studies were as follows: (1) ALS patients diagnosed with other neurodegenerative diseases such as Alzheimer's disease, Parkinson, and Huntington's disease; (2) reviews, case reports, animal experiments, and non-randomized controlled trials; (3) studies with missing data or repeated publications; (4) high-calorie therapy not being a heterogeneous intervention in the experimental group.

### Study selection

All retrieved studies were managed in the Note Express software. Firstly, duplicates were removed. Then, study screening was carried out by two independent researchers separately. The studies that did not meet the inclusion criteria were excluded by reading the titles and abstracts. Researchers downloaded the remaining studies and read the full text to determine whether they could meet the inclusion criteria. Any disagreement on the selection process would be resolved by consulting the third researcher (Table 2).

**Table 2:** Characteristics of the included studies

Study	Sample size (exp/con)	Mean age (exp/con)	Disease duration	Intervention/control	Course of treatment	Outcome(s)	Follow-up duration
Dorst (2022)	15/16/17/16	(61.5 ± 7.5)/(57.6 ± 7.0)/(59.9 ± 9.5)/(62.1 ± 10.5)	16.0 (9.0–35.0)/13.0 (9.0–25.0)/19.0 (10.5–26.5)/16.0 (9.0–26.8) m	HCFD/UHCFS/UHCCS/OC	4 weeks	AE; body weight; CNAQ; ULM; ALSFRS-R; ROADS; NfL	4 weeks
Wang (2022)	20/20	(57.45 ± 12.18)/(56.8 ± 11.52)	(13.25 ± 3.43)/(12.25 ± 2.94) y	HCS/outine diet	6 months	Body weight; BMI; TF; Alb; HGB; PA; TC; TG; LDL; HDL; TLC; survival rate	Every month till patient’s death
Silva (2010)	8/8	Not mentioned	Not mentioned	Whey protein/maltodextrin	16 weeks	Body weight; BMI; ALSFRS-R; CRP; Alb, PA, CK, Cre, urea, glucose, AST, ALT, TLC, PLT, Na, POT	Not mentioned
Dorst (2020)	28/32	62.4 ± 10.8	Not mentioned	HCFD/placebo	18 months	NfL	Not mentioned
Ludolph (2020)	103/104	(62.4 ± 11.0)/(62.4 ± 10.6)	15.5 (9.6–23.2)/15.4 (9.9–25.3) m	HCFD/placebo	18 months	Survival rate, AE; CNAQ; ALSFRS-R; ROADS; SVC; BMI	14 days
Wills (2014)	9/8/7	(57.5 ± 15.4)/(64.0 ± 6.9)/(63.2 ± 9.4)	10.9 (3.3–23.3)/16.7 (9.5–19.1)/14.9 (8.8–27.3)	HCD/HFD tube-feeding formula	4 months	Body weight; survival rate; AE; BMI; FVC; ALSFRS-R, TC; TG; LDL; HDL; glucose; Alb; CRP	1 month

*HCFD* high-caloric fatty diet, *UHCFS* ultrahigh-caloric fatty supplement, *UHCCS* ultrahigh-caloric carbohydrate-rich supplement, *OC* open control, *HCS* high-calorie supplements, *HCD* high-carbohydrate diet, *HFD* high-fat diet, *AE* adverse event, *CNAQ* Council of Nutrition Appetite Questionnaire, *ULM* Ulm Nutrition Questionnaire, *ALSFRS-R* ALS Functional Rating Scale-Revised, *ROADS* Rasch-Built Overall Amyotrophic Lateral Sclerosis Disability Scale, *NfL* serum neurofilament light chain, *BMI* body mass index, *TF* transferrin, *Alb* albumin, *HGB* hemoglobin, *PA* prealbumin, *TC* total cholesterol, *TG* triglyceride, *LDL* low-density lipoprotein, *HDL* high-density lipoprotein, *TLC* total lymphocyte count, *CRP* C-reactive protein, *CK* creatine kinase, *Cre* creatine, *AST* aspartate transaminase, *ALT* alanine transaminase, *PLT* platelets, *Na* sodium, *POT* potassium, *SVC* slow vital capacity, *FVC* forced vital capacity

### Data extraction

Two reviewers independently extracted the following items from included studies: (1) first author, publication year, the sample size for each group, mean age; (2) interventions, comparisons, treatment time, courses of disease; (3) primary outcome: adverse events, ALSFRS-R scores, and survival rate; secondary outcome: body weight, body mass index, and laboratory parameters including high-density lipoprotein, low-density lipoprotein, and total cholesterol. If the original study presented pre- and post-intervention means and standard deviations (SD), we would follow the formulas provided in the Cochrane Handbook for the Evaluation of Intervention Systems to calculate the mean and SD of the differences. If the data were presented as interquartile range or median, the formulas derived by Wan et al. [16] were used for conversion.

### Risk of bias and certainty of evidence assessment

Two blinded independent researchers used the Cochrane Risk of Bias Tool (ROB 2.0 (Centre for Evidence-Based Medicine Odense (CEBMO), Odense, DK)) [17] to evaluate the quality of included studies from five dimensions: (1) bias arising from the randomization process; (2) bias due to deviations from intended interventions; (3) bias due to missing outcome data; (4) bias in the measurement of the outcome; (5) bias in the selection of the reported result. Any disagreements were consulted with the third researcher for resolution.

Two independent researchers used the Grading of Recommendations, Assessment, Development and Evaluations (GRADE) [18] approach to assess the evidence's certainty. Firstly, the initial level of certainty was determined by different study types. If the original study was RCT, high certainty could be awarded, but the observational study was regarded as low certainty. Then, the evidence would be degraded if there existed a risk of bias, inconsistency, indirectness, imprecision, or publication bias. Finally, the levels of evidence were classified as high, moderate, low, and very low. All procedures were implemented in GRADEpro software.

### Statistical analysis

All statistical analyses were performed in STATA 15.0 software. We analyzed the data with risk ratios (RR) and 95% confidence interval (CI) for dichotomous variables. For continuous variables, MD and 95% CI were chosen to be analyzed. *I*^2^ statistics assess heterogeneity [19]. *I*^2^ > 75% is recognized as significant heterogeneity, 50% < *I*^2^ ≤ 75% as moderate heterogeneity, 25% < *I*^2^ ≤ 50% as low heterogeneity, and *I*^2^ ≤ 25% as homogeneity. We used the fixed-effects model if heterogeneity was low or homogeneous. Otherwise, we chose the random effects model. Sensitivity analysis was performed to detect the source of heterogeneity. Moreover, we used funnel plots and Egger’s regression test to assess the publication bias.

## Results

### Summary of included research

**Fig. 1:**
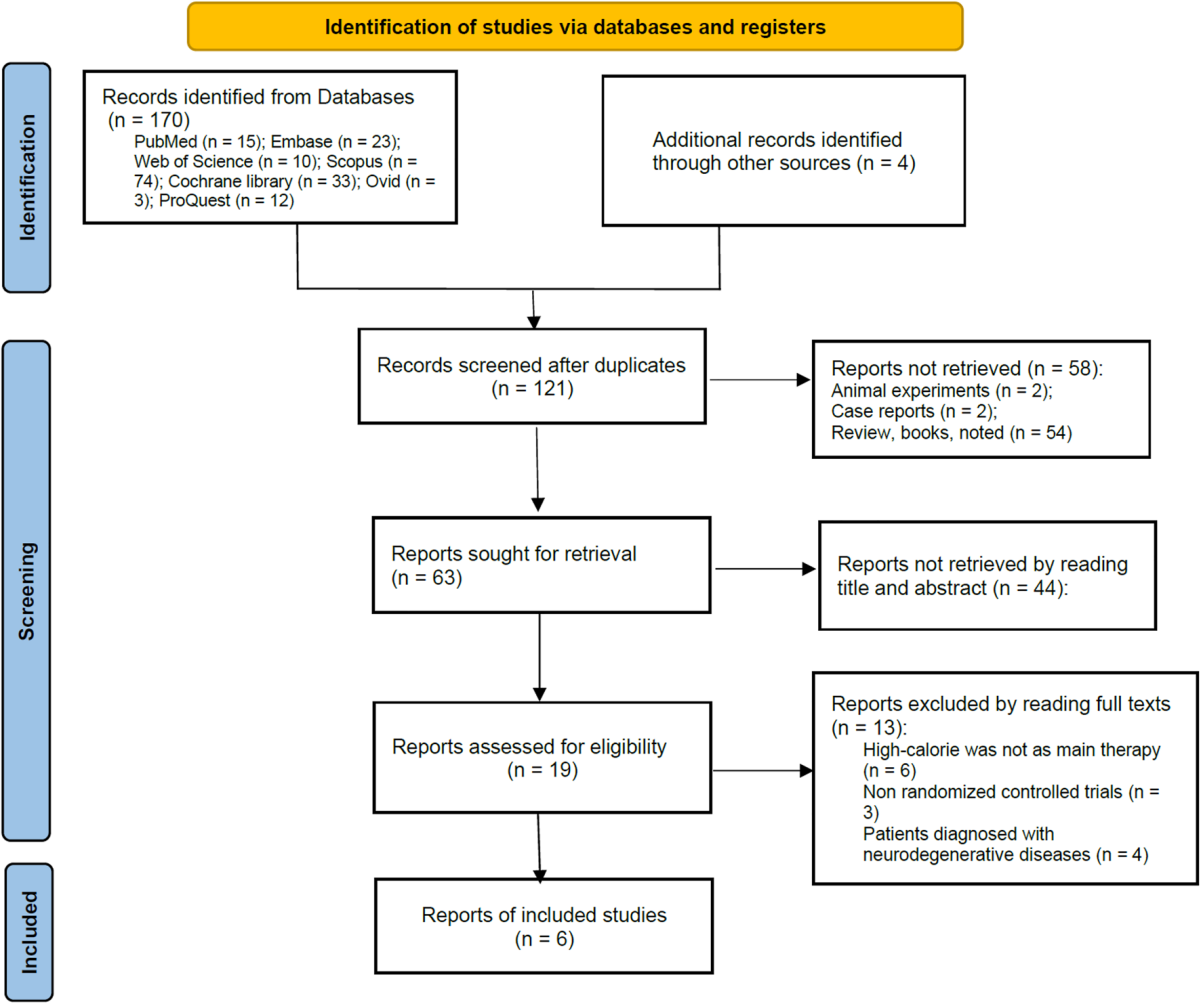
Flow diagram of the selection process

A total of 170 potential studies were retrieved from seven electronic databases based on the search strategy. After removing 49 duplicates, the remaining 121 studies were assessed for eligibility by reading the title and abstracts. We read the full texts of 19 potential studies for further review, and 6 RCTs involving 411 ALS patients were eligible for this meta-analysis. The flow diagram of the study selection is shown in Fig. 1.

The interventions varied slightly in the six included studies. Two studies treated ALS patients with high-fat therapy, one with high-protein diet, one was described as a high-calorie intervention only, one had both a high-carbohydrate and a high-fatty supplement group, and one divided the ALS patients into four groups with high-fat, ultrahigh-fat, ultrahigh-carbohydrate supplements and open control. Since this meta-analysis aims to evaluate the effectiveness and safety of high-calorie therapy, dose–response analysis was not included in our study. Of note, high-calorie therapy includes high carbohydrate, high fat, and high protein. The study of "Wills2014 (A)" refers to high-carbohydrate therapy, and "Wills2014 (B)" refers to high-fat therapy.

### Quality assessment and risk of bias

The included studies were assessed using the Cochrane Risk of Bias Tool 2.0 to assess the five domains of RCTs associated with the risk of bias while giving an overall bias. Based on the results, almost all included studies were assessed with low risk. However, Saibo wang2022 was not described in detail in the section on deviations from intended interventions, and therefore had some methodological concerns with the overall risk, the included studies were all RCTs with no missing data, and the reasons for missing visits were explained in the studies. The assessment process is shown in Table 3.

**Table 3:** Quality assessment and risk of bias

Study	Randomization process	Deviations from intended interventions	Missing outcome data	Measurement of the outcome	Selection of the reported result	Overall bias
Johannes Dorst (2022)	Low	Low	Low	Low	Low	Low
Saibo Wang (2022)	Low	Some concerns	Low	Low	Low	Some concerns
Luciano (2010)	Low	Low	Low	Low	Low	Low
Johannes Dorst (2020)	Low	Low	Low	Low	Low	Low
Albert (2020)	Low	Low	Low	Low	Low	Low
Anne (2014)	Low	Low	Low	Low	Low	Low

### Meta-analyses of outcomes

#### Primary outcome

(1) Adverse events (AEs).

**Fig. 2:**
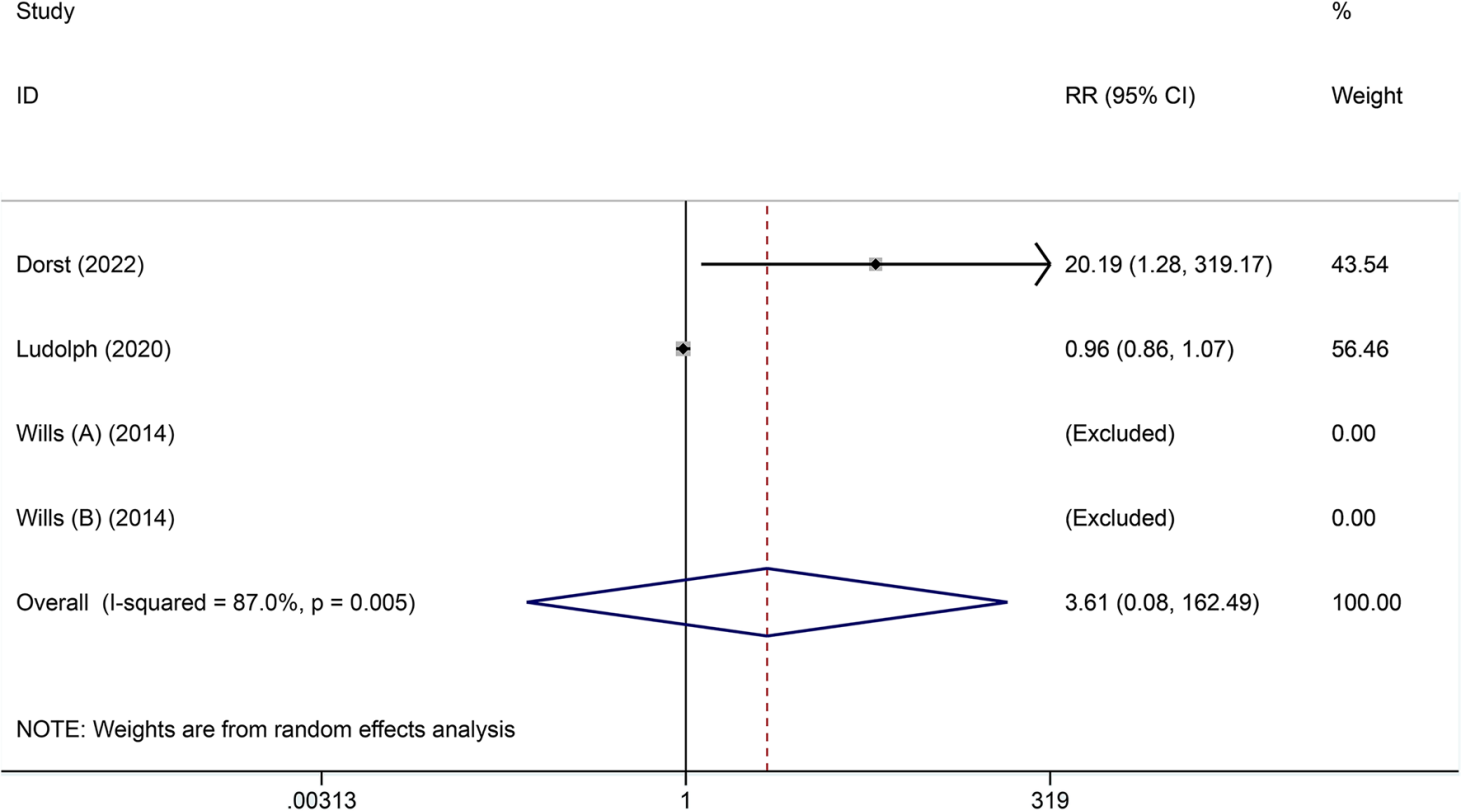
Forest plot of adverse events

Three included studies [20–22] reported the number of AEs, so they were subjected to meta-analysis. Random effects model was performed for significant heterogeneity detected (*I*^2^ = 87%). The meta-analysis results showed no difference between the high-calorie therapy and the control group regarding the number of AEs (RR = 3.61, 95% CI 0.08, 162.49, Fig. 2). Of note, it is reported that the main AEs of high-calorie therapy were temporary gastrointestinal symptoms (nausea, vomiting, diarrhea, constipation, abdominal pain), a feeling of fullness and loss of appetite, infections, pain in the extremities, cough, and dyspnea.

(2) ALSFRS-R scores.

**Fig. 3:**
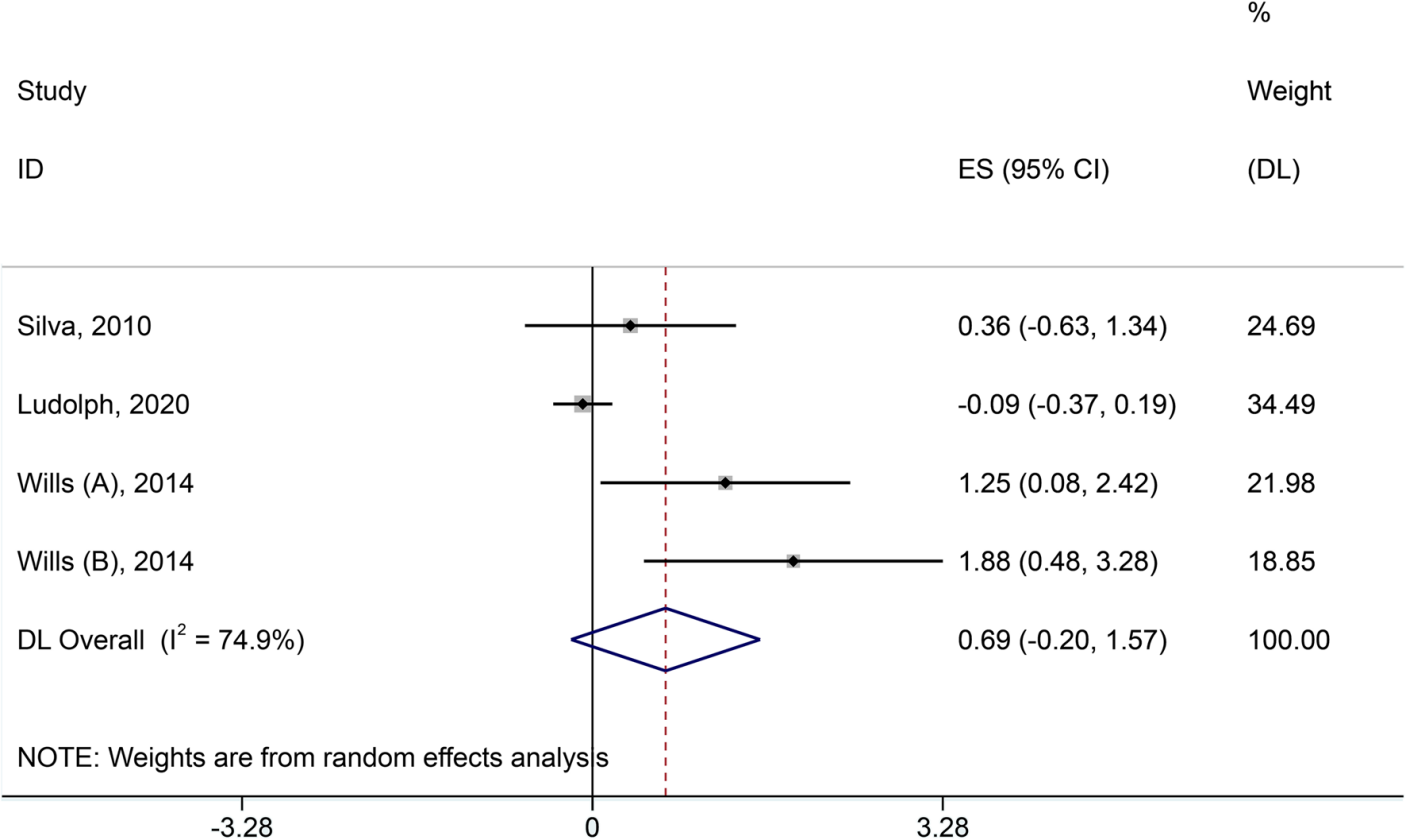
Forest plot of ALSFRS-R scores

Four included studies [20–23] reported ALSFRS-R scores, but Johannes Dorst et al. [20] did not disclose the raw data of ALSFRS-R scores in the original study. We failed to obtain the original data by connecting the authors. Hence, we excluded this research. Heterogeneity was apparent with *I*^2^ = 74.9%, *Q*-test *P* < 0.1. We chose the random effect model for this outcome analysis. The result showed no difference between the high-calorie therapy and control groups in the ALSFRS-R scores (SMD = 0.69, 95% CI − 0.2, 1.57, Fig. 3).

(3) Survival rate.

**Fig. 4:**
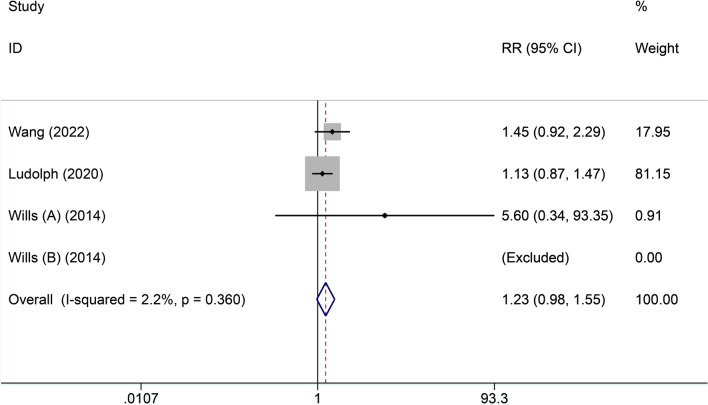
Forest plot of survival rates

Three included studies [21, 22, 24] reported the number of deaths. There was homogeneity among the three studies (*I*^2^ = 2.2%, Cochran's *Q* test = 0.36). Therefore, we chose a fixed-effects model. It did not show that high-calorie therapy had superiority in improving survival rate compared with the control group (RR = 1.23, 95% CI 0.98, 1.55, Fig. 4).

#### Secondary outcome

(1) Body weight.

**Fig. 5:**
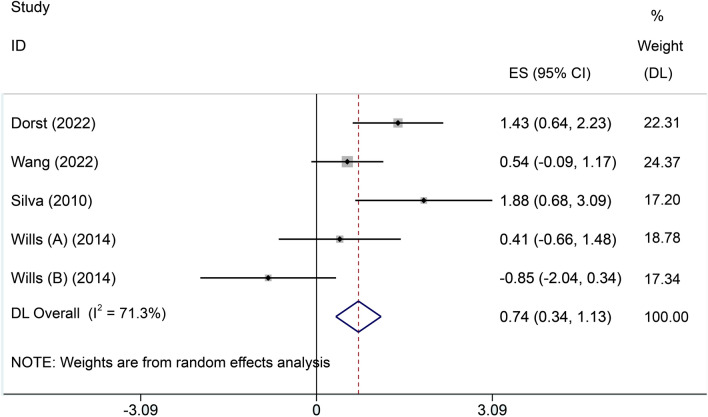
Forest plot of body weight

Four included studies[21, 22, 24] reported changes in body weight. An evident heterogeneity was detected among these four independent studies (*I*^2^ = 71.3%). We chose a random effect model for meta-analysis and the result showed that high-calorie group surpassed control groups in weight gain (SMD = 0.74, 95% CI 0.34, 1.13, Fig. 5).

(2) BMI

**Fig. 6:**
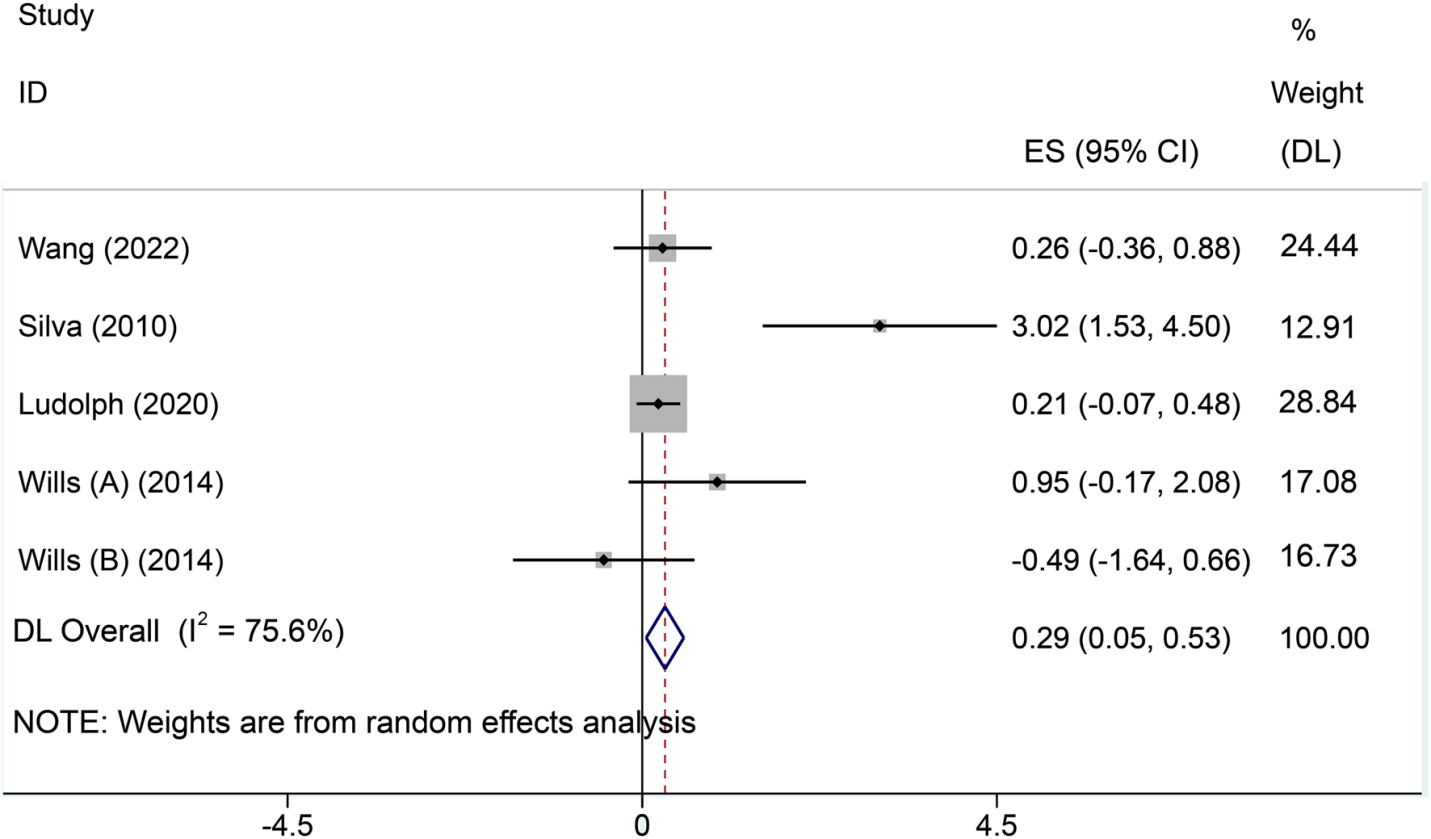
Forest plot of BMI

Four included studies[21–24] reported BMI. Significant heterogeneity was detected (*I*^2^ = 75.6%), so a random effects model was selected for meta-analysis. The results showed that high-calorie therapy increased BMI compared to that of controls (SMD = 0.29, 95% CI 0.05, 0.53, Fig. 6).

(3) Lipid profile.

**Fig. 7:**
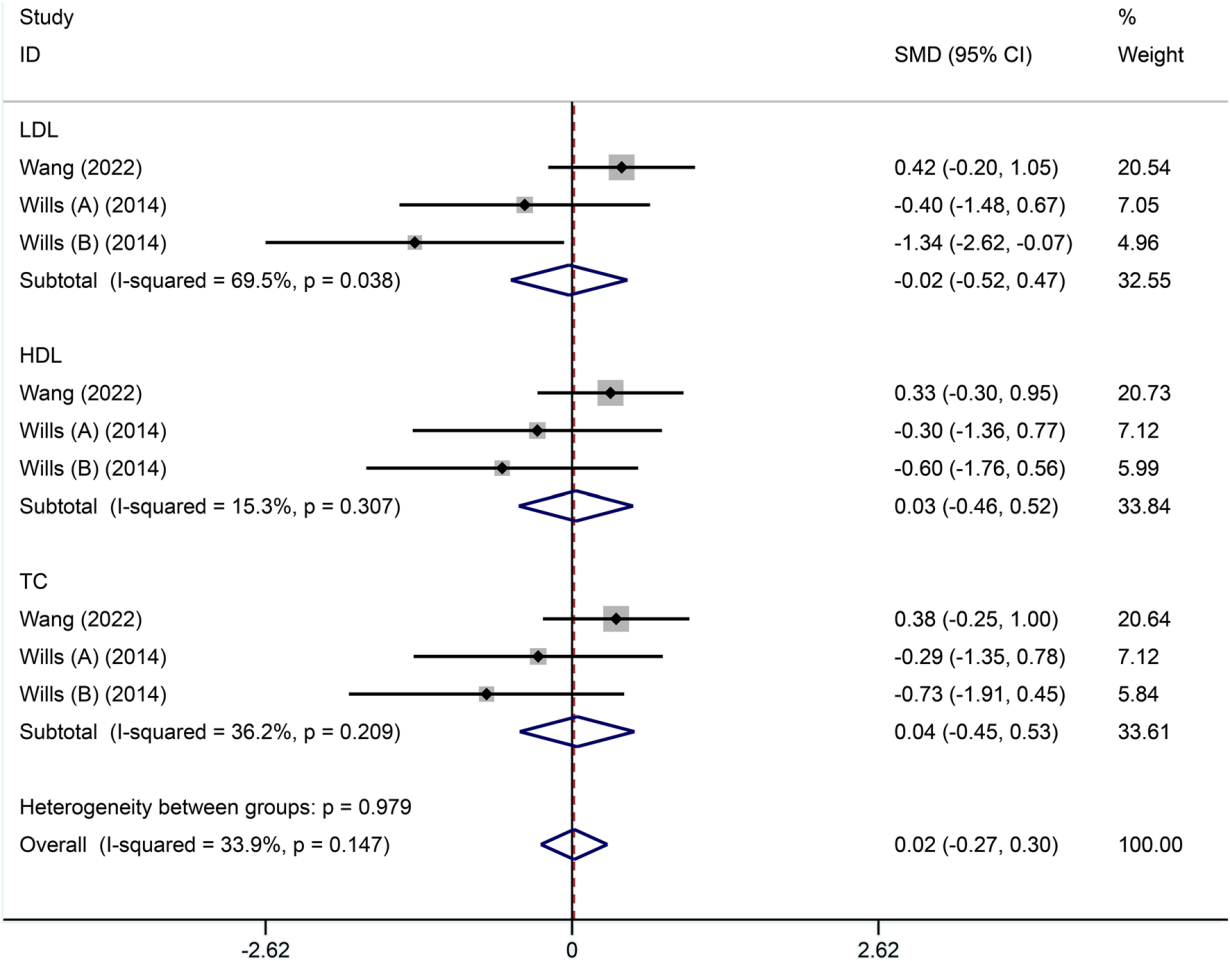
Forest plot of lipid profile

Two included studies[22, 24] reported on lipid profile parameters, including LDL, HDL, and TC. There was a homogeneity among all three indicators (LDL: *I*^2^ = 69.5%, HDL: *I*^2^ = 15.3%, TC: *I*^2^ = 36.2%). Therefore, the fixed-effects model was selected for meta-analysis in three indicators respectively, and the results showed no difference between the high-calorie therapy compared to the control groups in any of the three indicators of lipid profile (LDL: SMD = − 0.02, 95% CI − 0.52, 0.47; HDL: SMD = 0.03, 95% CI − 0.46, 0.52; TC: SMD = 0.04, 95% CI − 0.45, 0.53, Fig. 7).

(4) C-reactive protein (CRP).

**Fig. 8:**
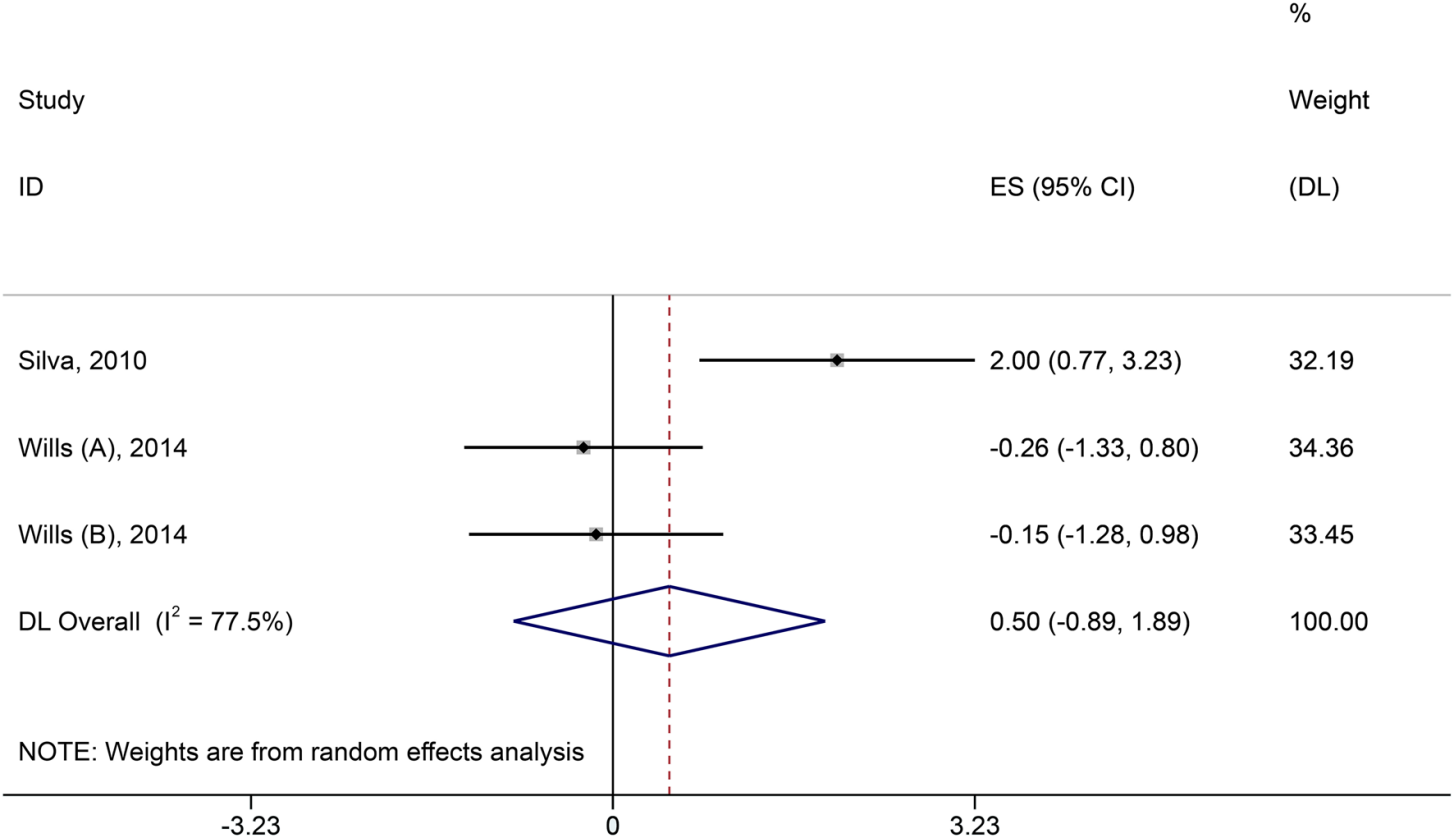
Forest plot of CRP

Two included studies reported on CRP indicators. We chose a random effects model for analysis because a significant heterogeneity was detected in two studies. The result showed no statistical difference between high-calorie therapy on CRP compared to controls (SMD = 0.5, 95% CI − 0.89, 1.89, Fig. 8).

### Subgroup analyses

We performed subgroup analyses based on different types of high-calorie therapies. Although only six RCTs were included in this meta-analysis, four studies treated ALS patients with high-fat therapies. One was with high-protein and one with high-calorie therapy without a detailed description. Therefore, we mainly performed a statistical analysis of whether high-fat therapy can be effective and safe for ALS patients. However, the results of outcomes, including AEs, ALSFRS-R, survival time, body weight, and BMI, showed no significance compared to the control groups, as shown in Appendix Fig. 10.

### GRADE analysis for the certainty of the evidence

**Fig. 9:**
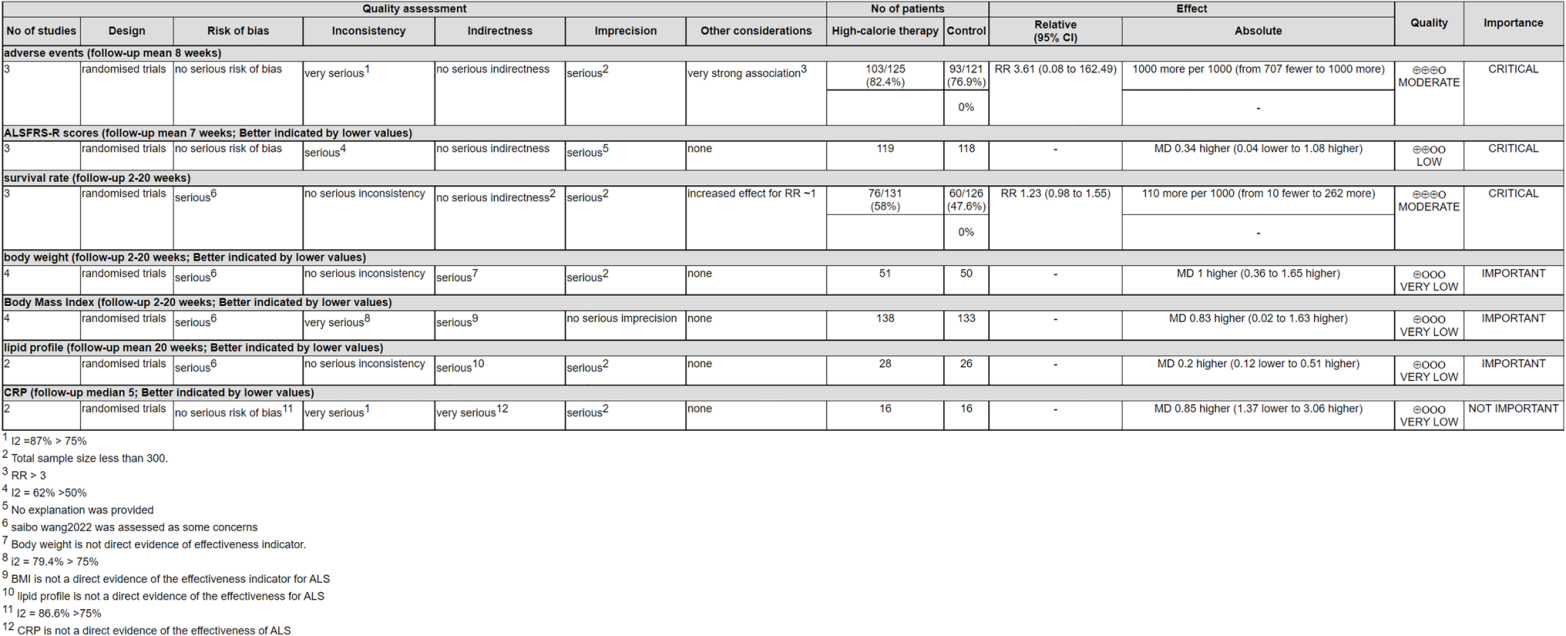
GRADE analysis and certainty of evidence

Although RCTs of included studies were considered the highest level of evidence, the quality evidence of outcomes still needed to be interpreted cautiously. The outcomes of adverse events and survival rate were assessed as moderate evidence because the domains of imprecision and inconsistency did not meet the criterion. The outcome of ALSFRS-R scores was defined as low evidence, because the heterogeneity between included studies was significant. Furthermore, body weight, BMI, lipid profile, and CRP were assessed as very low-quality evidence by being downgraded to the risk of bias, indirectness, and imprecision (Fig. 9).

## Discussion

The systematic review and meta-analysis focused on the safety and effectiveness of high-calorie therapy for treating amyotrophic lateral sclerosis patients. In this meta-analysis of six RCTs involving 370 individuals, high-calorie therapy increased body weight and BMI. Though there were, to some extent, improvements in the primary outcomes of ALSFRS-R scores and survival time, the pooled estimate did not achieve statistical significance. No severe adverse events were reported for the safety outcome, and common gastrointestinal reactions, such as diarrhea, nausea, and vomiting, were self-limiting and temporary. With the extensive reading of full texts of included studies, we revealed that the disease duration of patients was mainly in the range of 9–24 months. Therefore, we cautiously speculate that ALS patients with a disease duration of fewer than 2 years and milder symptoms who receive high-calorie therapy at an early stage may effectively gain weight and thus mitigate the progression of the disease.

Amyotrophic lateral sclerosis is a devastating neurodegenerative disease in which the leading cause of death is inadequate energy intake, malnutrition, and respiratory insufficiency due to complications of dysphagia. A direct association between nutritional status and ALS disease progression has been confirmed. Approximately, 16–55% of ALS patients are diagnosed with malnutrition, and most patients suffer weight loss from diagnosis. An independent prognostic factor linked to shorter survival is regarded as exacerbating muscle loss from denervation [25, 26]. Designing RCTs with stronger feasibility in terms of the debilitating nature of ALS is challenging. As part of a complementary and alternative intervention, high-calorie therapy, a kind of diet management, is feasible to have better compliance for ALS patients. The concept of high calorie includes nutrients with high fat, high carbohydrate, and high protein. We included RCTs in this meta-analysis as long as they used the above-mentioned three therapies. To date, neuroscientists and nutritionists have been dedicated to the role of high-calorie diets in ALS disease progression, and some observational studies have conducted high-calorie diets as an exposure factor. Researchers have also conducted RCTs, but no definitive conclusion exists among mutually independent studies on whether a high-calorie diet can slow disease progression. We systematically searched the databases and identified one systematic review without meta-analysis, which indicated that high-calorie therapy could gain body weight and BMI, prolong survival time, and improve functional status [27]. However, the conclusion from the small sample size was considerably mixed between the results of different studies. Indeed, the absence of meta-analysis may question the validity due to a lack of transparency [28]. Therefore, the results should be interpreted with caution.

To better evaluate which diet would have the best effect on ALS patients, we summarized the composition of dietary supplements in included studies. According to Table 4, all the dietary supplements studied comprised protein, carbohydrates, and fats. One was rich in vitamins, and after 12 months of follow-up, Saibo Wang et al. pointed out that the number of survivors in the experimental group was significantly superior to the number of survivors in the control group [24]. Xia et al. conducted Mendelian randomization of the relationship between dietary nutrients and ALS. Of note, they found that fat-soluble vitamins such as vitamin D and E delayed the progression of ALS and that unsaturated fatty acids were protective factors for ALS. In contrast, linoleic acid was a risk factor for ALS [29]. Less oral intake, potential hypermetabolic state, and progressive muscle atrophy resulted in malnutritional status and weight loss for ALS patients, associated with rapid progression. Emanuele et al. noted the significance of nutritional assessment at the earliest referral. When patients are diagnosed with ALS, adequate nutrition and restriction of pro-inflammatory food intake may help patients with weight maintenance or further weight gain, which can counteract the hypermetabolic state caused by ALS. Further nutritional research is warranted to provide a better understanding of the role of ALS in nutritional and metabolic abnormalities and explore the pathogenic mechanisms of ALS [30]. Boeun et al. conducted an observational study of nutritional intake and survival time in ALS with up to 6 years of follow-up, and they concluded that higher dose intake of fat and protein, especially from meat, in the early stage of the disease could prolong the survival time of ALS patients [31]. So far, there is still no definitive recommendation as to which diet is best for patients with MND. However, a narrative review of the published literature shows that researchers tend to recommend multivitamin-rich, high-protein, high-fat, and antioxidant foods for ALS patients, but not pro-inflammatory foods. Because one of the mechanistic hypotheses of the disease focuses on neuroinflammation, long-term consumption of pro-inflammatory foods may exacerbate neuroinflammation and worsen the disease.

**Table 4:** Composition of dietary supplements in included studies

Study	Composition of dietary supplements
Johannes Dorst (2022)	Protein, carbohydrate, saturated fat, sodium
Saibo Wang (2022)	Protein, carbohydrate, fat, α-linolenic acid, linoleic acid, dietary fiber, vitamin A, C, D, E, K1, B1, B2, B6, B12, niacin, folic acid, pantothenic acid, biotin, sodium, potassium, copper, magnesium, iron, zinc, manganese, calcium, phosphorus, iodine, chlorine, selenium, chromium, molybdenum, choline
Luciano (2010)	Whey protein
Johannes Dorst (2020)	Fat
Albert (2020)	Fat
Anne (2014)	Fat, omega-3 fatty acids eicosapentaenoic acid and γ-linolenic acid, protein

At present, the pathogenesis of ALS remains unknown. However, studies have found that energy metabolism disorders are common in ALS patients [32]. On the one hand, the metabolic pathway of the tricarboxylic acid cycle in ALS patients is damaged [33]. On the other hand, the structure and function of mitochondria are destroyed, resulting in a decrease in the production of adenosine triphosphate [34]. Adenosine monophosphate-activated protein kinase (AMPK) is an intracellular stress sensor that maintains intracellular energy homeostasis [35]. When activated, AMPK consumes glucose, promotes fatty acid oxidation, and inhibits new cholesterol, lipid, and protein synthesis [36]. Studies have found that by feeding a high-fat diet to SOD1^G93A^ mutant mice, the AMPK activity is inhibited, heat shock protein 70 (HSP70), as a downstream substance of the AMPK cascade pathway, is significantly increased, and HSP70, as a molecular chaperone, contributes to the correct folding of proteins and can prolong the survival time of mice [37, 38]. In summary, a high-fat diet may improve ALS survival time by inhibiting AMPK activity. With an extensive reading of studies relevant to the survival rate, it was found that Ludolph (2020)’s study with a sample size exceeding 100 per group significantly differed from the studies of Wang (2022) and Wills (2014), which had sample sizes of less than 30 per group. When we performed subgroup analysis by sample size exceeding 30 for each group, high-calorie therapy significantly improved patients' survival rates in studies with small samples (RR = 1.65, 95% CI 1.03, 2.66), and no heterogeneity was detected (*I*^2^ = 3%). Of note, RCTs with larger sample sizes are more stable for meta-analysis results, and small samples involving significant results should still be interpreted with caution. Interestingly, the high-fat therapy in Ludolph (2020) failed to confirm a significant difference in survival compared to the control group. However, stratified analysis by disease progression showed that the high-fat diet group had significantly prolonged survival in the subgroup of fast disease progression. It also prompted us that when high-fat therapy should be conducted, for what population, and how many calories to consume should be taken into account.

In this meta-analysis, six studies were retrieved according to inclusion criteria. Three of them were treated with high-fat therapy. One was a high-carbohydrate, one was a high-protein, and one was a high-calorie diet without a detailed description. For the safety outcome, adverse events in high-calorie therapy were temporary, and no statistical difference was detected compared to the control group. Survival rate and ALSFRS-R scores were defined as effective indicators for the effectiveness of outcomes. From the results of meta-analyses, no statistical significance was detected in these two indicators, accounting for a relatively short duration in the experiment or ALS patients who discontinued high-calorie therapy when they ended the experiment. In the meantime, we tried to conduct subgroup analyses with different interventions. In the subgroup of high-fat diets, three studies reported the adverse events and provided complete raw data for meta-analysis. However, the rest of the outcomes, including CNAQ, ALSFRS-R, and ROADS scores, were presented with descriptive analyses. Furthermore, the high-carbohydrate, high-protein, and high-calorie regimens were reported in only one study; thus, subgroup analyses could not be performed.

In summary, we performed a meta-analysis by including all randomized controlled trials currently studying high-calorie therapy for amyotrophic lateral sclerosis. We concluded that the therapy is effective for ALS patients' weight gain and BMI stabilization. Meanwhile, it is safe. Although survival rates and ALSFRS-R scores did not show superiority, this simple, low-cost, low-risk therapy is still worth investigating. Motor neuron disease is a multiorgan systemic disease, and ALS is one of its most common subtypes. There is mounting evidence from investigations demonstrating that it appears to be restricted to more than the central nervous system and affects systemic physiology. Furthermore, the clinical presentation is heterogeneous, which suggests that ALS is accompanied by a series of syndromes rather than one nosological entity. Amyotrophic lateral sclerosis is typically characterized by dysfunctional metabolism, and researchers are becoming more interested in using dietary interventions to treat neurological diseases [32, 39]. Because of the scarcity of treatment options for amyotrophic lateral sclerosis, more extensive, multicenter, long-course intervention randomized controlled trials should be conducted to explore the effectiveness of high-calorie therapy.

### Limitations

This study has some inherent limitations. First, though the RCT methodology is robust evidence, the pooled analyses based on small sample sizes studies were not stable. Second, the limited included studies made it impossible to perform a dose–response analysis of high calorie to determine which level would be better to alleviate the progression of the disease.

## Conclusion

The meta-analysis showed that high-calorie therapy was effective against a conventional diet regarding body weight and BMI gain, while no significant side effects occurred. However, no significant superiority was shown in survival time, ALSFRS-R score, CRP, and lipid profile. The level of GRADE evidence ranged from very low to moderate. The outcomes of survival time and ALSFRSR scores did not show statistical differences, which may be explained by the short duration of the intervention and the inability of patients to live with high-calorie therapy after the experiment. Nevertheless, further high-quality RCTs with long-term follow-ups are necessary to validate the efficacy and safety of high-calorie therapy for treating ALS.

## Figures and Tables

**Fig. 10:**
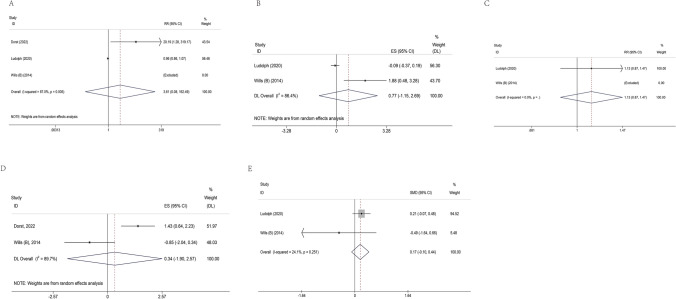
Subgroup analysis

## Data Availability

The data that support the findings of this study are available from the corresponding author, [Yan Zhao and Jing Zhou], upon reasonable request.

## Appendix

See Fig. 10.
